# Menstrual hygiene management among adolescent school girls in Taraba State, Nigeria

**DOI:** 10.4314/ahs.v21i2.45

**Published:** 2021-06

**Authors:** Esther Umahi Nnennaya, Sonnen Atinge, Somterimmam Paul Dogara, Rimande Joel Ubandoma

**Affiliations:** 1 Department of Public Health, Faculty of Health Sciences, Taraba State University Jalingo, Nigeria; 2 Taraba State Health Services Management Board, Jalingo, Taraba State, Nigeria

**Keywords:** Menstrual hygiene, adolescents, Taraba, Nigeria

## Abstract

**Background:**

The onset of menstruation denotes a landmark event in pubertal development of the adolescent girl. Lack of adequate knowledge and good menstrual hygiene management can have far reaching consequences on the girl's wellbeing, dignity and reproductive health.

**Objectives:**

This study assessed the menstrual knowledge and hygiene practices of adolescent school girls in Taraba State, Nigeria.

**Methods:**

A descriptive cross sectional study conducted among 297 adolescent school girls. Participants were selected using multistage sampling technique. A self-administered, structured questionnaire was used for data collection. The Statistical Package for Social Sciences (SPSS) version 22.0 was used for the analysis of data.

**Results:**

The mean age at menarche was 13.7 years (± 6.7). Over three-quarter (76.1%) of the girls knew about menstruation before experiencing it. Mothers (48.1%) were the leading source of information about menstruation to the students. The study found that 207 (69.7%) of respondents had good knowledge about menstruation while 171 (57.58%) had good menstrual hygiene management. Knowledge was significantly associated with good menstrual hygiene management (p<0.001).

**Conclusion:**

Knowledge of menstruation and hygienic practices during menstruation among the participants in the study was encouraging. Every adolescent girl should be equipped with the right knowledge and support for good menstrual hygiene management.

## Introduction

Adolescents form a sizeable proportion of the population and an important resource of any country. The World Health Organization recognizes adolescent group as comprising people between the ages of 10 and 19 years^1^. The onset of adolescence is usually associated with the commencement of puberty and the appearance of secondary sex characteristics^2^. Menstruation, a unique sign of sexual maturity occurring one or two years after the appearance of secondary sexual characteristics, is the periodic vaginal flow of blood that occurs with the shedding of the uterine mucosa^3^. The onset of menstruation denotes a landmark event in pubertal development of the adolescent girl.

Knowledge about menstruation and menstrual hygiene is critical to the dignity and well-being of girls and women in general^4^,^5^. Regardless of culture, age, and marital status, adolescents need basic, accurate and complete information as regards their body structure and functions, as well other sexual and reproductive health issues. Poorly informed choices and practices have potential for long-term deep negative effects on their reproductive health^6^. Conversely, accurate knowledge and hygienic practices during menstruation has impact on multiple areas across the sustainable development goals including health, education, gender equality, and water and sanitation^7^. Evidence showed that poor personal hygiene and unsafe sanitary conditions have primarily resulted in gynecological problems among the adolescent girls^8^. A preponderance of cases of infections due to lack of hygiene during menstruation has been reported^9^,^10^,^11^. Hidden micro-organisms and vaginal infections have been reported to be caused by frequent use of unclean napkins or the improperly dried cloth napkins before its reuse^8^. Furthermore, the growing urban waste problem in developing countries was reported to be partly due to inappropriate disposal of absorbents used during menstruation^12^.

Several studies have indicated that many girls receive little or no premenarcheal information^13^, ^14^. A study in India reported that 70% of the girls had not heard about menstruation before attainment of menarche^15^. In Southeast Nigeria, another study found that only 44% of the adolescent girls had premenarcheal training, which resulted to inappropriate menstrual experiences and poorer menstrual hygiene practice^13^. Girls most often ask mothers, other female family members as well as peers to obtain information about menstruation^16^. Frequently, these people are either not well equipped to fill gaps in girls' knowledge or do not feel comfortable discussing menstruation due to religious or cultural restrictions^17^. Though a normal physiologic process, menstruation is still associated with negative feelings and notions in some individuals and communities hence the culture of silence and shame surrounding it and sexuality in general^18^. In Nepal, for instance, some rural families still observe an ancient tradition of banishing grls and women to sheds when they have their menstruation^19^. A study revealed that even in high income countries with adequate facilities and information, menstruation is considered shameful and embarrassing among adolescent girls^20^.

Challenges faced by adolescent girls in low-and middle income countries in relation to effective menstrual hygiene management include lack of access to clean, effective absorbents; inadequate facilities to change, clean and dispose absorbents; lack of access to soap and water; and lack of privacy^21^–^24^. In the absence of suitable and affordable menstrual care products, some women and girls resort to using unhygienic and inappropriate products such as newspapers, old rags, dried leaves, or socks to collect menstrual blood and manage their products^23^,^24^. According to a report in East Africa, 4 out of 5 girls lack access to sanitary pads and related health education^15^. A study in Mali reported the use of old cotton fabric (pagnes) to absorb menstrual blood among adolescent girls^25^. Also, from Ethiopia a research showed that 35.4% of students used sanitary napkins, 55.6% used homemade cloths, and 9% used underwear as absorbent materials for menstrual blood^26^. In Nigeria, a survey conducted in northwestern State of Kaduna showed that only 37% of women age 15–49 have everything they need such as clean materials, a facility, pain medication, and places to dispose of used products for proper menstrual hygiene^27^. UNICEF however, recommends the use of clean menstrual hygiene management (MHM) materials to absorb or collect blood that can be changed in privacy as often as necessary for the duration of the menstrual period^5^. Furthermore, adolescent girls are expected to use soap and water to bath at least twice a day, wash their underwear and change their clothing daily and to have access to facilities to dispose of used menstrual management material^21^.

This study assessed menstruation knowledge and hygienic practices during menstruation among adolescents in Taraba State. The results will be beneficial in planning programs for improving adolescents' knowledge level of good menstrual hygiene management with regard to their life processes and advancement of their quality of life.

## Methods

### Study Design, Study Area and Population

The study was descriptive cross sectional conducted in Jalingo town. Jalingo is both the capital city of Taraba State as well as a Local Government Area (L.G.A.) in the State. It is the most developed and most densely populated L.G.A. in Taraba. It is surrounded by Ardo-Kola, Yaro, and Lau Local Government Areas. It is situated at 8.88° North Latitude, 11.37° East Longitude and 351 meters' elevation above sea level. According 2016 projected population, it had 187,500 persons with 32,050 between the ages 10 – 19 (28). The population is heterogeneous in nature with numerous ethnic groups with varied historical and socio cultural backgrounds. There are a total of 24 secondary schools in Jalingo, 12 public and 12 private. The study population was adolescent school girls between the ages of 10 – 19 years in selected secondary schools in Jalingo. All adolescents who had not attained menarche were excluded from the study

### Sample Size and Sampling Procedure

The sample size for the study was estimated using the simplified formula for proportions: n = N/1+ N (e)2 (29), where n is the unknown sample size, N is the total population (1014), e is the level of precision which is 0.05 with 95% confidence interval. The calculated sample size was 287 which was increased by 10% to account for non-response rate. The participants were selected using a multi-stage sampling technique: the first stage involved simple random sampling, by lottery method of 4 secondary (2 private and 2 government) schools from 24 secondary schools in Jalingo LGA. The selected schools were Howai, Government Day, Jetters Montessori and Kenneth Foundation Group of schools. In the second stage, 27 classes out of 54 were selected using the proportionate to size sampling method based on the female students' population in each school. Consequently, 10, 3, 3, and 11 classes were sampled from Howai, Jetters Montessori, Kenneth Foundation Group of schools and Government day secondary schools which have 130, 25, 20 and 140 adolescent girls respectively. In the third stage, respondents in the sampled classes were selected using systematic sampling technique with a sampling interval of 3 after selecting the first respondent by simple random sampling.

### Data Collection and Management

A pre-tested structured self-administered questionnaire was used to obtain information from the participants. The questionnaire collected information on the sociodemographic data of the respondents, knowledge about menstruation and menstrual hygiene practices. All questionnaires were screened for completeness, and a total of 297 were coded, scored and entered into the computer. The Statistical Package for Social Sciences (SPSS) version 22.0 was used for the analysis of data. The findings were presented in tables and charts. Significant associations were evaluated using Chi-squared test and level of significance was considered at 0.05.

An 8-point knowledge scale was used to measure the respondents' knowledge. A correct knowledge attracted one point while a wrong knowledge was zero. A score of ≤ 4 points was considered poor while a score ≥ 5 was considered good in determining composite score for knowledge variable. Menstrual hygiene practice was determined using a 7-point practice scale. A good menstrual hygiene practice attracted a score of 1 while the score for a poor practice was zero. Scores of < 4 and ≥ 4 points were considered poor and good practices respectively

### Ethical Considerations

Ethical clearance to carry out the study was obtained from the Taraba State Ministry of Education Jalingo. The principals of each school also gave permission for the study. An informed verbal consent was obtained from each student and only those who gave consent were interviewed. Anonymity and confidentiality was observed as the questionnaires bore no names or students' ID numbers. Their right to refuse participation was respected.

## Results

### Socio-Demographic Characteristics

A total of 297 respondents participated in the study. The largest age group was 13 to 15 years, 146 (49.2%) followed by 16 – 19 years 136 (45.8%). Majority of the respondents (213; 71.7%) were from the different minority tribes in Taraba State (Mumuye, Jukun, Chamba, Itchen, etc). With respect to their religious affiliation, 256 (86.2%) were of the Christians faith while 36 (12.1) were Muslims. Two hundred and eight (70%) of the respondents attained menarche at between the age group of 13 – 15 years, with mean age at menarche 13.7 (±6.7). Two hundred and twenty (74.1%) of the respondent's mothers had SSCE as their highest educational qualification while 77 (25.9%) had tertiary education (Table 1).

**Table 1:** Socio-demographic Characteristics of Respondents

Variables	Frequency (n = 297)	Percent (%)
**Age Group (in years)**		
10–12	15	5.1
13–15	146	49.2
16–19	136	45.8
**Ethnicity**		
Hausa/Fulani	54	18.2
Igbo	25	8.4
Yoruba	5	1.7
Others	213	71.7
**Religion**		
Christian	256	86.2
Islam	36	12.1
Traditional	5	1.7
**Class**		
JSS	65	21.9
SSS	232	78.1
**Live with**		
Both Parents	146	49.2
Mother only	66	22.2
Relatives	76	25.6
Others	9	3.0
**Age at First menarche**		
10–12	58	19.5
13–15	208	70.0
16–18	31	10.4
Mean age at menarche	**13.7 (± 6.7)**	
**Mother's Education**		
No formal Education	39	13.2
Primary	52	17.5
Secondary	129	43.4
Tertiary	77	25.9
**Mother's Occupation**		
Housewife	81	27.3
Trading	81	27.3
Civil Servant	73	24.6
Farmer	62	20.9

### Sources of Menstrual Knowledge

**Figure 1:**
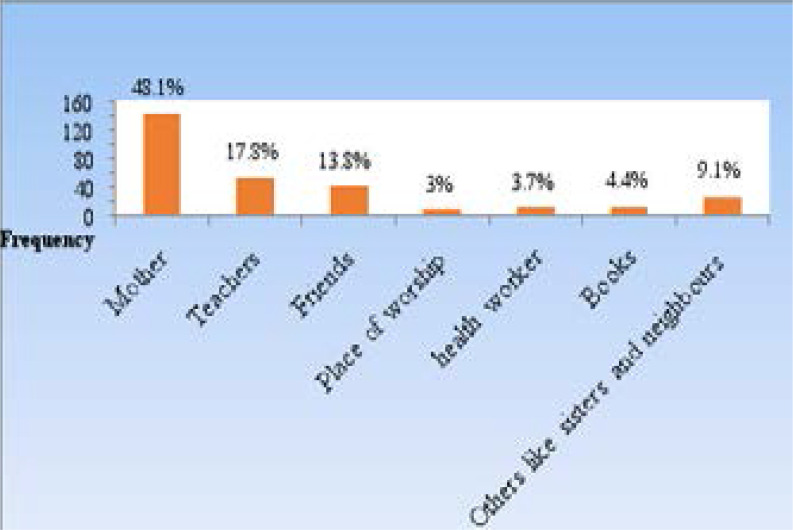
Showing adolescent school girls sources of knowledge about menstruation

The leading source of menstrual knowledge were mothers, 142 (48.1%), followed by teachers, 55 (17.8%), friends 40 (13.8%) and others like sisters, 24 (9.1%) (Figure 1)

### Knowledge of menstruation

Three-quarter of respondents 226 (76.1%) had heard about menstruation before menarche. Also, 112 (38%), which is the total of respondents that said ‘no’ and ‘not sure’, did not know that menstruation is the passage of blood from the uterus every 28 – 30 days. One hundred and twenty one (40.7%) of the respondents thought that other materials other than sanitary pads can be used as absorbents for menstruation.

### Menstrual Hygiene Practices

Less than half” (127; 42.8%) of the respondents used sanitary pads, the majority (244; 82.2%) bathed twice daily; 186 (62.6%) cleaned external genitalia with soap and water; 103 (34.7%) collected pads, toilet rolls and cotton wools as the case may be after usage and disposed of in the latrine; majority; 129 (43.4%) changed their absorbents three times a day (Table 3).

**Table 3:** Respondents Menstrual Hygiene Practices

Variables	Frequency (n = 297)	Percent (%)
**Type of absorbent used**		
New piece of cloth	89	30.0
Old piece of cloth	32	10.8
Sanitary pad	127	42.8
Toilet paper	26	8.8
Cotton wool	23	7.7
**Bathing during menstruation**		
Once	32	10.8
Twice	244	82.2
Never	21	7.1
**Cleaning of external genitalia**		
Soap and water	186	62.6
Water and antiseptic	57	19.2
Water only	54	18.2
**Disposal of absorbents**		
Burning	81	27.3
Dustbin	76	25.6
Latrines or toilets	103	34.7
Burying	37	12.5
**No of times absorbents are** **changed per day**		
Once	23	7.7
Twice	93	31.3
Thrice	129	43.4
>3 times	52	17.5
**Health Seeking behavior:**		
Takes pain medications		
Yes	120	40.5
No	138	46.5
Not sure	39	13.1
**Premenstrual exercise to prevent pain**		
Yes	116	39.1
No	139	46.8
Not sure	42	14.1

### Reasons for not using Sanitary Pads

**Figure 2:**
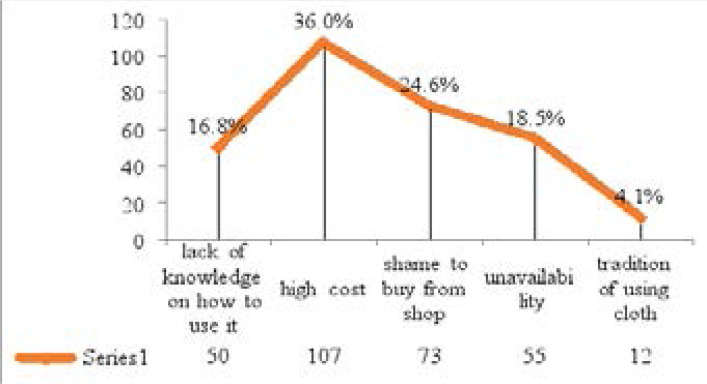
Respondents' reasons for not using sanitary pad

One hundred and seven (36%) of the respondents reported high cost of disposable pads, 73 (24.6%) shame to buy from shop, 55 (18.5%) reported unavailability and 50 (16.8%) lack of knowledge on how to use it (Figure 2).

### Association between adolescents' knowledge level and menstrual hygiene practices

There was a statistically significant association between knowledge of menstruation processes and menstrual hygiene practices (p<0.001). A higher proportion of adolescents with good knowledge 171 (82.61%) had good menstrual hygiene practices. In contrast, respondents who had poor knowledge all had poor practice 90 (100%) [Table 4].

**Table 4:** Association between adolescents' level of knowledge and menstrual hygiene practices

Level of knowledge	Menstrual hygiene Practices			
	Poor n (%)	Good n (%)	Total n (%)	*χ2 (p value)*
Poor	90 (100%)	0 (0)	90 (100)	175.25 (p<0.001)
Good	36 (17.39%)	171 (82.61%)	207 (100)	

## Discussion

A total of 297 adolescent girls participated in the study. Seventy percent of respondents experienced first menstruation between ages 13–15 years with mean age at menarche been 13.7 ± 6.7. Minority ethnic groups in Nigeria which are more than 20 in Taraba State made up the largest group, 213 (71%) of the total study population.

Awareness about menstruation in this study was appreciable. Over three-quarter (76.1%) of the girls knew about menstruation before experiencing it. This is similar with findings from other studies in Nigeria and elsewhere^7^,^30^,^31^. The high level of awareness may not be unconnected with the study location, being an urban area. Most previous studies were also conducted in cities where exposure and access to information is generally higher either through parents who are likely to be more educated, or through the mass or social media. Perhaps, studies done in rural settings will report lower awareness than these figures usually found from city populations.

The study showed that mothers were the leading source of information about menstruation to the students. This is in agreement with results of studies within and outside Nigeria^7^,^30^–^33^. The finding is however contrary to those from Ethiopia and Egypt^28^,^33^ where majority of the participants (67.8%) got information about menstruation from friends, followed by the mass media (57%). A possible explanation for this difference may be that girls from those climes discuss menstruation issues with their peers more openly. Generally, it is agreed that the home environment should provide the primary information on sexual education, particularly the mothers to the girl child. What is sometimes worrying about this source is that the depth of knowledge passed by the mothers is a function of their level of education, communication skills as well as socio-cultural prescriptions and proscriptions. This therefore often leads to incomplete or incorrect information about the subject. The finding of a high proportion of the girls with poor knowledge of the source of menstruation, process and its timing, despite high awareness in this study may be an attestation to this fact. A study by Water-Aid^32^ in Nepal also echoed this concern where they reported that the knowledge about menstruation that large majority of the girls had before attaining menarche, and later too, was not correct. But as seen in this study and several others^7^,^30^–^33^ those who should fill this knowledge gap by virtue of their educational knowledge and training are usually not major sources of this knowledge.

Menstrual hygiene management during menstruation is of considerable importance as it can affect women's health by increasing their vulnerability to infections of the urinary tract and the perineum^9^,^11^. This study found that sanitary pads were the most used absorbent 127 (42.8%) among the adolescents. Though this is similar with two separate studies done in Kano, North-western Nigeria, the latter found percentages that are more than twice higher (92.2% and 93.8% respectively) than this case^7^,^34^. This higher use from Kano could be explained from the fact that the secondary schools selected were within Kano metropolis where significant number of students belong to more affluent homes who could afford to buy the menstrual pads for their children as compared to Jalingo, a relatively young and only growing town. The fact that high cost of sanitary pads was cited most (36%) as the reason for non-use attest to this explanation. Elsewhere in other cities like Benin and Ife, sanitary pads were also the absorbent used by majority of the students^30^, ^35^. This is unlike the Water-Aid study in Nepal where most of the girls made use of new pieces of cloth^32^. Similarly, less than onehalf (43.4%) of the respondents changed absorbents at least 3 times daily during menstruation. This is about the same with 42.5% reported in Ile-Ife, South-western Nigeria^30^. It is however lower than that reported in Sokoto, North-western Nigeria^36^ where 70% of respondents changed absorbents at least 3 times daily.

On method of disposal, this study revealed that most adolescents disposed of used absorbents in the latrines or toilets (34.7%), by burning (27.3%), in dustbins (25.6%). This is at variance with the report from Kano^7^ where dustbin was the major disposal method (73.9%) then followed by the disposal in the toilet (14.7%). The finding in this study is surprisingly similar to the one in Ife, in Southern Nigeria, where about the same proportion (35.17%) used the pit latrine as the major disposal method and equally followed too by burning (32.6%)^30^. During menstruation, most respondents (82.7%) bathed at least twice daily similar to 84.1% in Ife and 83.3% in Benin City^30^, ^35^. This contrasts the finding from a study in Nepal where 57% of the participants bathed only daily and 43% on alternate days when menstruating^37^. On a sharp contrast, a report from India revealed that as high as 62.3% of females abstained from taking their bath during menstruation due to the belief that bathing would either stop the menstrual flow or increase its intensity^9^. Cleanliness of the external genitalia during menstruation was satisfactory. Majority of the girls (62.6%) used soap and water which is the ideal healthwise, and 19.2% used water and antiseptic. A similar finding was reported in India where 57.3% also used both soap and water for cleaning the external genitalia. The significant few (18.2%) who used water only possibly did so either as a result of lack of knowledge or unavailability of soap or both. A significant association was found between good knowledge and practice of menstrual hygiene management (p<0.001). Other studies in Nigeria are also in agreement with this finding^11^,^23^. In many instances in real life including health behaviour, this is the normal expectation, a positive correlation between knowledge and practice. It fails to be true only when other variables such as enabling factors confound the direction of outcome.

## Conclusion

There was good knowledge and practice of menstruation and menstrual hygiene among the adolescent school girls. Sanitary pads were the most used menstrual absorbent. Most people who used other materials did so because of cost of affording sanitary pads. Majority of the adolescents properly disposed of menstrual absorbents. Promotion of menstrual hygiene among adolescents should be maintained. Governments and organizations should make sanitary pads and other menstrual hygiene management facilities and services affordable to every adolescent girl.

## Figures and Tables

**Table 2:** Knowledge of Respondents Regarding Menstruation

Variables	Frequency (n=297)	Percent
**Heard about menstruation before menarche**		
Yes	226	76.1
No	37	12.5
Not Sure	34	11.4
**Normal menstrual bleeding duration**		
3–7 days	84	28.3
5–7 days	44	14.8
1–3 days	169	56.9
**Menstruation is passage of blood** **from the uterus**		
Yes	185	62.3
No	30	10.1
Not Sure	82	27.6
**Menstruation results from shedding** **of the walls of the uterus**		
Yes	167	56.2
No	20	6.7
Not sure	110	37.0
**Menstruation comes with pain and ill health**		
Yes	271	91.2
No	13	4.4
Not sure	13	4.4
**During menstruation, personal hygiene** **is important**		
Yes	277	93.3
No	10	3.4
Not sure	10	3.4
**Menstruation is bad blood washed** **away from the body**		
Yes	238	80.1
No	38	12.8
Not sure	21	7.1
**I know when to expect my menses**		
Yes	212	71.4
No	35	11.8
Not sure	50	16.8
**Rags, cloths and tissue paper can be used** **as absorbent**		
Yes	121	40.7
No	127	42.8
Not sure	49	16.5
